# Trends in Nonfatal and Fatal Overdoses Involving Benzodiazepines — 38 States and the District of Columbia, 2019–2020

**DOI:** 10.15585/mmwr.mm7034a2

**Published:** 2021-08-27

**Authors:** Stephen Liu, Julie O’Donnell, R. Matt Gladden, Londell McGlone, Farnaz Chowdhury

**Affiliations:** ^1^Division of Overdose Prevention, National Center for Injury Prevention and Control, CDC; ^2^Peers and Partners, Inc., Seattle, Washington.

Nonfatal and fatal drug overdoses increased overall from 2019 to 2020 (*1*).* Illicit benzodiazepines (e.g., etizolam, flualprazolam, and flubromazolam)^†^ were increasingly detected among postmortem and clinical samples in 2020, often with opioids,^§^ and might have contributed to overall increases in drug overdoses. Availability of recent multistate trend data on nonfatal benzodiazepine-involved overdoses and involvement of illicit benzodiazepines in overdoses is limited. This data gap was addressed by analyzing annual and quarterly trends in suspected benzodiazepine-involved nonfatal overdoses^¶^ treated in emergency departments (EDs) (benzodiazepine overdose ED visits) during January 2019–December 2020 (32 states and the District of Columbia [DC]) and benzodiazepine-involved overdose deaths (benzodiazepine deaths), which include both illicit and prescription benzodiazepines, during January 2019–June 2020 (23 states) from CDC’s Overdose Data to Action (OD2A) program. From 2019 to 2020, benzodiazepine overdose ED visits per 100,000 ED visits increased (23.7%), both with opioid involvement (34.4%) and without (21.0%). From April–June 2019 to April–June 2020, overall benzodiazepine deaths increased 42.9% (from 1,004 to 1,435), prescription benzodiazepine deaths increased 21.8% (from 921 to 1,122), and illicit benzodiazepine deaths increased 519.6% (from 51 to 316). During January–June 2020, most (92.7%) benzodiazepine deaths also involved opioids, mainly illicitly manufactured fentanyls (IMFs) (66.7%). Improving naloxone availability and enhancing treatment access for persons using benzodiazepines and opioids and calling emergency services for overdoses involving benzodiazepines and opioids, coupled with primary prevention of drug use and misuse, could reduce morbidity and mortality.

CDC’s OD2A program collects data on unintentional and undetermined intent drug overdoses: 1) nonfatal overdoses treated in EDs from the Drug Overdose Surveillance and Epidemiology (DOSE) system, and 2) overdose deaths from the State Unintentional Drug Overdose Reporting System (SUDORS).** Benzodiazepine overdose ED visits during January 2019–December 2020 were identified from 33 DOSE jurisdictions (32 states and DC)^††^ submitting data to the National Syndromic Surveillance Program. Benzodiazepine and opioid overdose ED visits were identified using diagnosis codes and chief complaint text fields.^§§^ Only EDs consistently reporting informative data^¶¶^ during January 2019–December 2020 were included to ensure valid trend analyses. Relative rate percentage changes for benzodiazepine overdose ED visits per 100,000 ED visits were calculated by quarter (Q1: January–March, Q2: April–June, Q3: July–September, and Q4: October–December) and stratified by opioid involvement.***

Benzodiazepine deaths were identified from 23 states^†††^ participating in SUDORS. States obtained data from death certificates and medical examiner and coroner reports, including complete postmortem toxicology testing results. Benzodiazepine deaths during January 2019–June 2020, percentages involving any opioids and opioid type (heroin, IMFs, or prescription opioids),^§§§^ and percentage change in deaths, were calculated by quarter.

Demographic characteristics of persons experiencing nonfatal and fatal benzodiazepine overdoses, and specific drug co-involvement for benzodiazepine deaths, were described using the most recent period of available data (benzodiazepine overdose ED visits: January–December 2020, benzodiazepine deaths: January–June 2020). Benzodiazepine deaths were stratified by benzodiazepine type (prescription or illicit). Chi-square tests were used for pairwise comparisons; p-values <0.05 were considered statistically significant. Analyses were conducted using SAS (version 9.4; SAS Institute). This activity was reviewed by CDC and conducted consistent with applicable federal law and CDC policy.^¶¶¶^

During January 2019–December 2020, 117 million ED visits reported in the 33 jurisdictions (72% of total ED visits) qualified for inclusion in analyses. Among these, 31,377 benzodiazepine overdose ED visits were identified, including 15,547 in 2019 and 15,830 in 2020; 6,883 (21.9%) also involved opioids. The highest number of benzodiazepine overdose ED visits occurred in Q3 2020 (4,181) (Figure 1).

**FIGURE 1:**
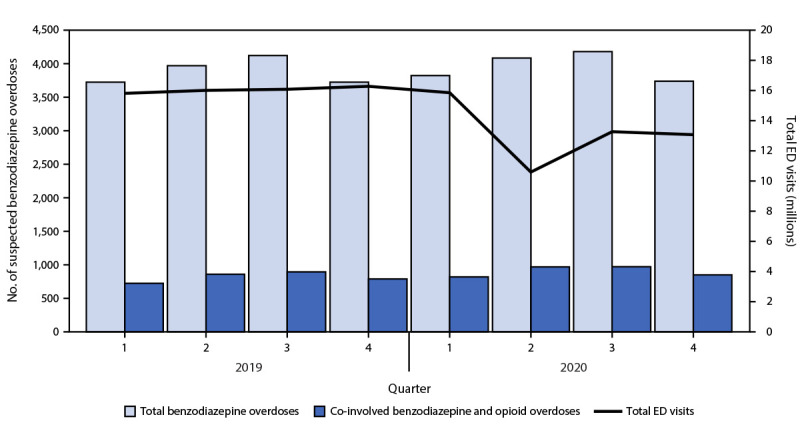
Number of benzodiazepine overdose and co-involved opioid overdose emergency department visits, by quarter — Drug Overdose Surveillance and Epidemiology, 32 States* and the District of Columbia, 2019–2020^†^^,^^§^^,^^¶^ **Abbreviations:** ED = emergency department; Q = quarter. * Alabama, Arizona, Arkansas, Connecticut, Delaware, Florida, Georgia, Illinois, Kansas, Kentucky, Louisiana, Maine, Maryland, Mississippi, Missouri, Montana, Nevada, New Jersey, New Mexico, New York, North Carolina, Ohio, Oregon, Pennsylvania, Rhode Island, South Carolina, Tennessee, Utah, Virginia, Washington, West Virginia, and Wisconsin. ^†^ All analyses were restricted to facilities with a coefficient of variation ≤45 and average weekly discharge diagnosis informative ≥75% during the study period, including consistently reporting facilities with consistent data quality. ^§^ Relative rate changes from 2019 to 2020 were calculated based on total benzodiazepine overdose visits per 100,000 ED visits (15,547/64,200,344 and 15,830/52,816,815, respectively; 23.7% increase); benzodiazepine overdose visits co-involving opioids (3,271/64,200,344 and 3,612/52,816,815, respectively; 34.4% increase); and those with no opioid co-involvement (12,276/64,200,344 and 12,218/52,816,815, respectively; 21.0% increase). ^¶^ Relative rates increased from Q4 2019 to Q4 2020 for total benzodiazepine overdose visits by 24.9% (3,727/16,279,535 and 3,739/13,080,832, respectively), those co-involving opioids by 34.0% (791/16,279,535 and 851/13,080,832, respectively), and those without opioids by 22.5% (2,936/16,279,535 and 2,888/13,080,832, respectively).

In 2020, benzodiazepine overdose ED visits more often involved females (51.5%) and persons aged 25–34 years (20.9%); nearly one quarter (22.8%) also involved opioids (Table). From 2019 to 2020, benzodiazepine overdose ED visits per 100,000 ED visits increased 23.7%, from 24.22 in 2019 to 29.97 in 2020, with larger rate increases among ED visits involving opioids (34.4% [from 5.09 to 6.84 per 100,000]) compared with those without opioids (21.0% [from 19.12 to 23.13 per 100,000]). Rates for any benzodiazepine ED visits were 24.9% higher in Q4 2020 (28.58) compared with those in Q4 2019 (22.89), as were rates for ED visits also involving opioids (34.0% increase from 4.86 in 2019 to 6.51 in 2020), and those without opioids (22.5% increase from 18.03 in 2019 to 22.08 in 2020) (Figure 1). All relative rate changes were statistically significant.

**TABLE:** Demographics of persons experiencing nonfatal and fatal benzodiazepine overdoses and specific drug involvement in fatal benzodiazepine overdoses — 38 States and the District of Columbia, 2020*

Characteristic	No. (%)			
	DOSE data: nonfatal benzodiazepine ODs, Q1–Q4 2020	SUDORS data: fatal benzodiazepine ODs, Q1–Q2 2020		
	Any benzodiazepine	Prescription	Illicit	Any benzodiazepine^†^
	N = 15,830	N = 2,174	N = 532	N = 2,721
**Sex**				
Male	7,655 (48.4)	1,288 (59.2)	382 (71.8)	1,690 (62.1)
Female	8,144 (51.5)	886 (40.8)	150 (28.2)	1,031 (37.9)
Unknown	31 (0.2)	0 (—)	0 (—)	0 (—)
**Age group, yrs**				
Median (IQR), yrs	38 (26–56)	41 (32–53)	33 (26–46)	40 (31–52)
0–14	545 (3.4)	—^§^	0 (—)	—^§^
15–24	2,928 (18.5)	155 (7.1)	111 (20.9)	261 (9.6)
25–34	3,302 (20.9)	513 (23.6)	175 (32.9)	687 (25.2)
35–44	2,666 (16.8)	606 (27.9)	108 (20.3)	707 (26.0)
45–54	2,128 (13.4)	440 (20.2)	75 (14.1)	526 (19.3)
55–64	2,255 (14.2)	372 (17.1)	53 (10.0)	437 (16.1)
≥65	1,961 (12.4)	86 (4.0)	10 (1.9)	101 (3.7)
Unknown/Missing	45 (0.3)	—^§^	0 (—)	—^§^
**Race/Ethnicity**				
White, non-Hispanic	N/A	1,762 (81.0)	349 (65.6)	2,116 (77.8)
Black, non-Hispanic	N/A	202 (9.3)	110 (20.7)	319 (11.7)
Other, non-Hispanic	N/A	43 (2.0)	—^§^	58 (2.1)
Hispanic	N/A	148 (6.8)	52 (9.8)	204 (7.5)
Unknown/Missing	N/A	19 (0.9)	—^§^	24 (0.9)
**Drug involvement**				
**Any opioid** ^¶^	3,612 (22.8)	2,025 (93.1)	493 (92.7)	2,523 (92.7)
Heroin	675 (4.3)	460 (21.2)	145 (27.3)	596 (21.9)
IMFs**	N/A	1,421 (65.4)	417 (78.4)	1,815 (66.7)
Illicit opioids^††^	N/A	1,542 (70.9)	459 (86.3)	1,973 (72.5)
Prescription opioids^§§^	N/A	922 (42.4)	117 (22.0)	1,026 (37.7)
Prescription and illicit opioids	N/A	452 (20.8)	83 (15.6)	518 (19.0)
**Prescription benzodiazepines**	N/A	2,174 (100)	79 (14.8)	2,174 (79.9)
Alprazolam	N/A	1,224 (56.3)	52 (9.8)	1,224 (45.0)
Other	N/A	1,166 (53.6)	32 (6.0)	1,166 (42.9)
**Illicit benzodiazepines**	N/A	79 (3.6)	532 (100)	532 (19.6)
Etizolam	N/A	35 (1.6)	151 (28.4)	151 (5.5)
Flualprazolam	N/A	41 (1.9)	376 (70.7)	376 (13.8)
Other	N/A	—^§^	20 (3.8)	20 (0.7)

**Abbreviations:** DOSE = Drug Overdose Surveillance and Epidemiology; IMFs = illicitly manufactured fentanyls; IQR = interquartile range; N/A = data not available; OD = overdose; Q = quarter; SUDORS = State Unintentional Drug Overdose Reporting System.

* Nonfatal benzodiazepine overdose data were from 32 states and the District of Columbia for overdose emergency department visits during January 1, 2020–December 31, 2020. Fatal benzodiazepine overdose data were from 23 states for deaths during January 1, 2020–June 30, 2020. ^†^ Numbers of any benzodiazepines fatal ODs will not reflect the sum of prescription plus illicit benzodiazepine fatal ODs because some deaths involved both, and some deaths had a generic listing of benzodiazepine involvement; therefore, the prescription or illicit status could not be classified. ^§^ Dashes indicate cell data suppressed because cell contains one to nine cases or to prevent calculation of other suppressed cells. ^¶^ Comparing prescription benzodiazepine overdose deaths to illicit benzodiazepine overdose deaths, the percent co-involvement of any opioids was not statistically significantly different at p<0.05; all other comparisons were statistically significantly different (sex, age, race/ethnicity, co-involvement of heroin, IMFs, illicit opioids, prescriptions opioids, prescription and illicit opioids, and benzodiazepine type). Because prescription/illicit benzodiazepine deaths were not mutually exclusive, chi-square testing was performed after excluding 79 deaths with both prescription and illicit benzodiazepine involvement; however, percentages of each demographic category and with each substance co-involvement were similar to the nonmutually exclusive categorizations.

** IMFs include fentanyl and illicit fentanyl analogs.

^††^ Illicit opioids include heroin, IMFs, and other non-fentanyl illicit synthetic opioids (e.g., isotonitazene).

^§§^ Prescription opioids include alfentanil, buprenorphine, codeine, hydrocodone, hydromorphone, levorphanol, loperamide, meperidine, methadone, morphine, noscapine, oxycodone, oxymorphone, pentazocine, prescription fentanyl, propoxyphene, remifentanil, sufentanil, tapentadol, and tramadol.

Benzodiazepines were involved in 6,982 (16.8%) of 41,496 overdose deaths during January 2019–June 2020 reported by 23 states, with opioids involved in 6,384 (91.4%) benzodiazepine deaths. During the first 6 months of 2020, a total of 2,721 overdose deaths involved any benzodiazepine, 2,174 involved prescription benzodiazepines, and 532 involved illicit benzodiazepines (Table). Compared with prescription benzodiazepine overdose decedents, higher percentages of illicit benzodiazepine overdose decedents were male (71.8% versus 59.2%); Black, non-Hispanic (20.7% versus 9.3%); and younger (53.8% versus 30.7% aged 15–34 years); and a lower percentage was non-Hispanic White (65.6% versus 81.0%). Almost all benzodiazepine deaths during January–June 2020 also involved opioids (92.7%) and often involved IMFs (66.7%). Illicit benzodiazepine deaths more often involved IMFs than did prescription benzodiazepine deaths (78.4% versus 65.4%), and less often involved prescription opioids (22.0% versus 42.4%).

The number of benzodiazepine deaths increased 42.9% from Q2 2019 (1,004) through Q2 2020 (1,435), with increases in both prescription (from 921 to 1,122; 21.8% increase) and illicit (from 51 to 316; 519.6% increase) benzodiazepine deaths (Figure 2). During this time, co-involvement of IMFs in benzodiazepine deaths increased 25.4%, from 56.7% to 71.1%.

**FIGURE 2:**
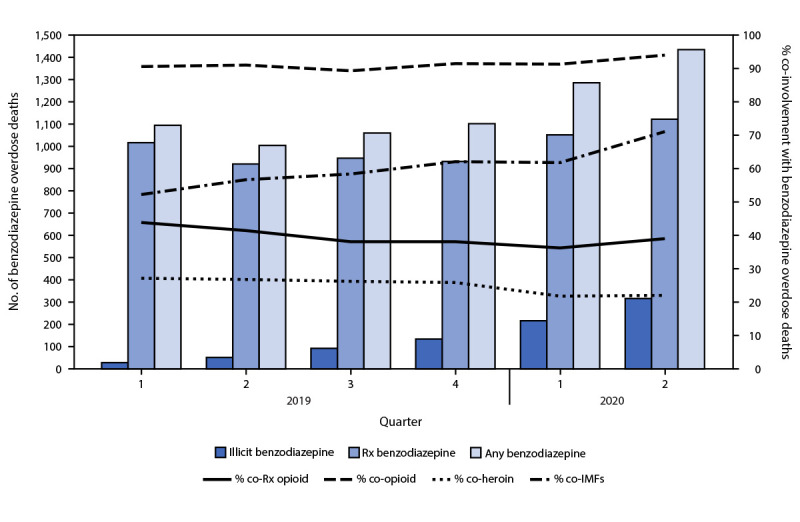
Benzodiazepine Overdose Deaths with Opioid Co-involvement — State Unintentional Drug Overdose Reporting System, 23 States,* January 2019–June 2020^†^ **Abbreviations:** co = co-involved; IMFs = illicitly manufactured fentanyls; Q = quarter; Rx = prescription. * Alaska, Connecticut, Delaware, Georgia, Illinois, Maine, Massachusetts, Minnesota, Missouri, Nevada, New Hampshire, New Mexico, North Carolina, Oklahoma, Pennsylvania, Rhode Island, Tennessee, Utah, Vermont, Virginia, Washington, West Virginia, and Wisconsin. ^†^ IMFs include fentanyl and illicit fentanyl analogs.

## Discussion

This is the first multistate report to examine recent trends in both nonfatal and fatal benzodiazepine overdoses. Three concerning trends during 2019–2020 were identified: 1) increases in both nonfatal and fatal overdoses involving benzodiazepines and opioids; 2) marked increases in illicit benzodiazepine deaths, although overdose deaths involving prescription benzodiazepines still far outnumber those involving illicit benzodiazepines; and 3) increases in nonfatal benzodiazepine overdoses not involving opioids. In 2016, the CDC Opioid Prescribing Guideline discouraged co-prescribing opioids and benzodiazepines,**** and the Food and Drug Administration imposed its most prominent warning on all benzodiazepine medications,^††††^ describing the risks of use with opioids. Despite progress in reducing co-prescribing before 2019 (*2*), this study suggests a reversal in the decline in benzodiazepine deaths from 2017 to 2019,^§§§§^ driven in part by increasing involvement of IMFs in benzodiazepine deaths and influxes of illicit benzodiazepines, likely indicating simultaneous use of nonprescribed opioids and benzodiazepines.

During 2019–2020, benzodiazepine deaths (both prescription and illicit) were characterized by high and increasing co-involvement of IMFs, a trend documented as early as during 2017–2018 (*3*). Substantial increases in the supply of IMFs during January 2013–June 2020,^¶¶¶¶^ coupled with the high potency and rapid absorption of IMFs (*4*), which increase overdose risk above that of heroin and prescription opioids, is likely a principal driver of fatal benzodiazepine and IMF overdose. The largest increase in IMF involvement among benzodiazepine deaths occurred in 2020 between Q1 and Q2, possibly reflecting altered drug use patterns that increased overdose risk (e.g., decreased naloxone access); and possible drug supply disruptions; during the COVID-19 pandemic (*5*). Although the much greater involvement of opioids in benzodiazepine deaths (91.4%) compared with benzodiazepine overdose ED visits (21.9%) underscores the dangers of co-use, increases in opioid involvement among benzodiazepine ED visits (34.4% increase) throughout 2020 might be an early indicator of continued and amplified increases in morbidity and mortality related to benzodiazepine and opioid co-use.

Other factors accelerating increases in benzodiazepine deaths involving opioids are rapid increases in supply and co-use of illicit benzodiazepines among persons using illicit opioids, especially IMFs. Whereas law enforcement reports of diverted prescription benzodiazepines declined from 2015 through June 2020, reports of illicit benzodiazepines (particularly etizolam and flualprazolam) surged during that period, indicating increased availability (*6*).***** Reductions in benzodiazepine and opioid co-prescribing must be coupled with efforts to disrupt and reduce the availability of and harms associated with concurrent use of illicit benzodiazepines and IMFs.

Although rates of ED visits for mental health conditions increased during 2019–2020 (*1*), benzodiazepine prescriptions remained relatively stable during January 2019–May 2020, with a transient spike in March 2020 indicating, per recommendations, increases in the availability of medications on hand because of stay-at-home orders to slow the spread of COVID-19 (*7*). However, the increases in benzodiazepine overdose ED visit rates, including those without opioids, raise concerns about increased misuse and warrant further investigation. Because benzodiazepine use is less likely to result in fatal overdose without use of opioids or other depressants (*6*), tracking nonfatal benzodiazepine overdoses is critical to tracking benzodiazepine misuse trends.

The findings in this report are subject to at least five limitations. First, jurisdictions included in nonfatal and fatal overdose analyses are not nationally representative and differ from each other, limiting the extent to which trends can be compared. Second, full toxicology results for nonfatal overdoses were not available, and opioid and benzodiazepine involvement in nonfatal overdoses is likely underestimated because comprehensive toxicology testing of persons treated for overdoses varies within and across EDs, and hospital discharge codes with drug specific information might be unavailable (*8*). Third, despite only including consistently reporting facilities, ED visits decreased sharply after implementation of COVID-19 mitigation measures in March 2020, which might inflate rate increases (*9*). Fourth, four states (Illinois, Missouri, Pennsylvania, and Wisconsin) reported overdose deaths from varying subsets of counties. Results were similar with and without these states. Finally, postmortem toxicology testing and drug involvement determination vary over time and across states, potentially affecting detection of specific drugs involved in deaths.

Increases in benzodiazepine overdose ED visits throughout 2020, coupled with increases in illicit benzodiazepine deaths since 2019, highlight the need to enhance efforts to mitigate harm from simultaneously using benzodiazepines and opioids and monitor the magnitude and persistence of increases in illicit benzodiazepine deaths. Persons who co-use opioids and benzodiazepines might be less likely to receive medications for opioid use disorder than persons using opioids only (*10*); therefore, efforts to increase treatment access should be enhanced. Expansion of naloxone availability and rapid naloxone administration should be encouraged for overdoses involving benzodiazepines and opioids because naloxone reverses opioid overdoses irrespective of benzodiazepine presence. However, educational efforts should emphasize the dangers of using illicit benzodiazepines, especially in combination with opioids, and the importance of calling 9-1-1 even after naloxone administration, because benzodiazepine overdose symptoms are unaffected by naloxone and might require additional medical treatment. These efforts, complemented by broader primary prevention of drug use and misuse, could prevent drug overdose morbidity and mortality.

**Summary**

**What is already known about this topic?**

Benzodiazepine-involved overdose deaths decreased during 2017–2019; however, since 2019, the illicit benzodiazepine supply increased.

**What is added by this report?**

From 2019 to 2020, benzodiazepine overdose visits per 100,000 emergency department visits increased (23.7%), both with (34.4%) and without (21.0%) opioid co-involvement. From April–June 2019 to April–June 2020, prescription and illicit benzodiazepine-involved overdose deaths increased 21.8% and 519.6%, respectively. During January–June 2020, 92.7% of benzodiazepine-involved deaths also involved opioids, and 66.7% involved illicitly manufactured fentanyls.

**What are the implications for public health practice?**

Improving naloxone availability and enhancing treatment access for persons using benzodiazepines and opioids and calling emergency services for overdoses involving benzodiazepines and opioids, coupled with primary prevention of drug use and misuse, could reduce morbidity and mortality.

## Footnotes

*
https://www.cdc.gov/nchs/nvss/vsrr/drug-overdose-data.htm

† “Illicit benzodiazepines” refers to benzodiazepines that are not marketed in the United States for medical purposes; “prescription benzodiazepines” refers to those that are marketed as prescription drugs in the United States (but does not imply that the decedent had a prescription for that benzodiazepine; prescription benzodiazepines might be diverted). https://www.who.int/medicines/access/controlled-substances/Final_Etizolam.pdf; https://www.npsdiscovery.org/wp-content/uploads/2019/12/Public-Alert_Flualprazolam_NPS-Discovery_120519.pdf; https://www.who.int/docs/default-source/controlled-substances/43rd-ecdd/final-flubromazolam-a.pdf?sfvrsn

§
https://www.npsdiscovery.org/reports/trend-reports/

¶ Analyses were intended to include nonfatal overdose visits with unintentional and undetermined intents. ED visits resulting in death were not excluded but accounted for < 0.5% of total benzodiazepine overdose ED visits in ESSENCE during the study period.

** https://www.cdc.gov/drugoverdose/od2a/index.html; https://www.cdc.gov/drugoverdose/data/nonfatal/

†† Alabama, Arizona, Arkansas, Connecticut, Delaware, District of Columbia, Florida, Georgia, Illinois, Kansas, Kentucky, Louisiana, Maine, Maryland, Mississippi, Missouri, Montana, Nevada, New Jersey, New Mexico, New York, North Carolina, Ohio, Oregon, Pennsylvania, Rhode Island, South Carolina, Tennessee, Utah, Virginia, Washington, West Virginia, and Wisconsin.

§§ https://www.cdc.gov/drugoverdose/data/nonfatal/case.html; https://knowledgerepository.syndromicsurveillance.org/cdc-benzodiazepine-v1

¶¶ Some ED facilities might have started reporting to National Syndromic Surveillance Program during the study period, and others experienced reporting disruptions. Analyses were performed among approximately 62% of EDs submitting data to National Syndromic Surveillance Program that had relatively stable trends based on a coefficient of variation ≤45 and average weekly discharge diagnosis informative ≥75%.

*** Both nonfatal and fatal overdoses often involve multiple drugs; therefore, estimates for drugs are not mutually exclusive. For example, an overdose co-involving opioid and benzodiazepine would be counted as both an opioid and benzodiazepine overdose.

††† Alaska, Connecticut, Delaware, Georgia, Illinois, Maine, Massachusetts, Minnesota, Missouri, Nevada, New Hampshire, New Mexico, North Carolina, Oklahoma, Pennsylvania, Rhode Island, Tennessee, Utah, Vermont, Virginia, Washington, West Virginia, and Wisconsin.

§§§ Drugs coded as prescription opioids were alfentanil, buprenorphine, codeine, hydrocodone, hydromorphone, levorphanol, loperamide, meperidine, methadone, morphine, noscapine, oxycodone, oxymorphone, pentazocine, prescription fentanyl, propoxyphene, remifentanil, sufentanil, tapentadol, and tramadol. IMFs include illicitly manufactured fentanyl and illicit fentanyl analogs. Fentanyl was classified as likely illicitly manufactured or likely prescription using toxicology, scene, and witness evidence. In the absence of sufficient evidence to classify fentanyl as illicit or prescription (<7% of deaths involving fentanyl), it was classified as illicit because most fentanyl overdose deaths involve illicit fentanyl.

¶¶¶ 45 C.F.R. part 46.102(l)(2), 21 C.F.R. part 56; 42 U.S.C. Sect. 241(d); 5 U.S.C. Sect. 552a; 44 U.S.C. Sect. 3501 et seq.

****
https://www.cdc.gov/mmwr/volumes/65/rr/rr6501e1.htm?CDC_AA_refVal

†††† https://www.fda.gov/drugs/drug-safety-and-availability/fda-drug-safety-communication-fda-warns-about-serious-risks-and-death-when-combining-opioid-pain-or; https://www.fda.gov/drugs/drug-safety-and-availability/fda-requiring-boxed-warning-updated-improve-safe-use-benzodiazepine-drug-class

§§§§ https://www.drugabuse.gov/drug-topics/trends-statistics/overdose-death-rates; https://wonder.cdc.gov/

¶¶¶¶
https://www.nflis.deadiversion.usdoj.gov/nflisdata/docs/13915NFLISdrugMidYear2020.pdf

*****
https://www.nflis.deadiversion.usdoj.gov/nflisdata/docs/13915NFLISdrugMidYear2020.pdf
